# Supplementary material for: Asthma-associated genetic variants induce IL33 differential expression through an enhancer-blocking regulatory region
Source: Nat Commun. 2021 Oct 21;12:6115. doi: 10.1038/s41467-021-26347-z (PMC8531453; doi:10.1038/s41467-021-26347-z)
Supplement: Supplementary file 1 — Supplementary Information [file 41467_2021_26347_MOESM1_ESM.pdf]

Supplementary Information

Asthma-associated variants induce *IL33* differential expression through a novel regulatory region

Ivy Aneas[1]*^, Donna C. Decker[2]*, Chanie L. Howard[3], Débora R. Sobreira[1], Noboru J. Sakabe[1], Kelly M. Blaine[2], Michelle M. Stein[1], Cara L. Hrusch[2], Lindsey E. Montefiori[1], Juan Tena[4], Kevin M. Magnaye[1], Selene M. Clay[1], James E. Gern[5], Daniel J. Jackson[5], Matthew C. Altman[6], Edward T. Naureckas[2], Douglas K. Hogarth[2], Steven R. White[2], Jose Luis Gomez-Skarmeta[4], Nathan Schoetler[2], Carole Ober[1], Anne I. Sperling[2,3]*^, Marcelo A. Nobrega[1]

Author affiliations:
1- Department of Human Genetics, University of Chicago, Chicago, IL 60637, USA.
2- Department of Medicine, Section of Pulmonary and Critical Care Medicine, University of Chicago, Chicago, IL 60637, USA.
3- Committee on Immunology, University of Chicago, Chicago IL 60637
4- Centro Andaluz de Biología del Desarrollo (CSIC/UPO/JA), Universidad Pablo de Olavide, Seville 41013, Spain.
5- Department of Pediatrics, University of Wisconsin School of Medicine and Public Health, Madison, WI 53726, USA
6- Division of Allergy and Infectious Diseases, Department of Medicine, University of Washington, Seattle, WA 98195, USA
*These authors contributed equally to this study

^ Corresponding Authors:

Supplementary Figure 1-8

Supplementary Table 1-8

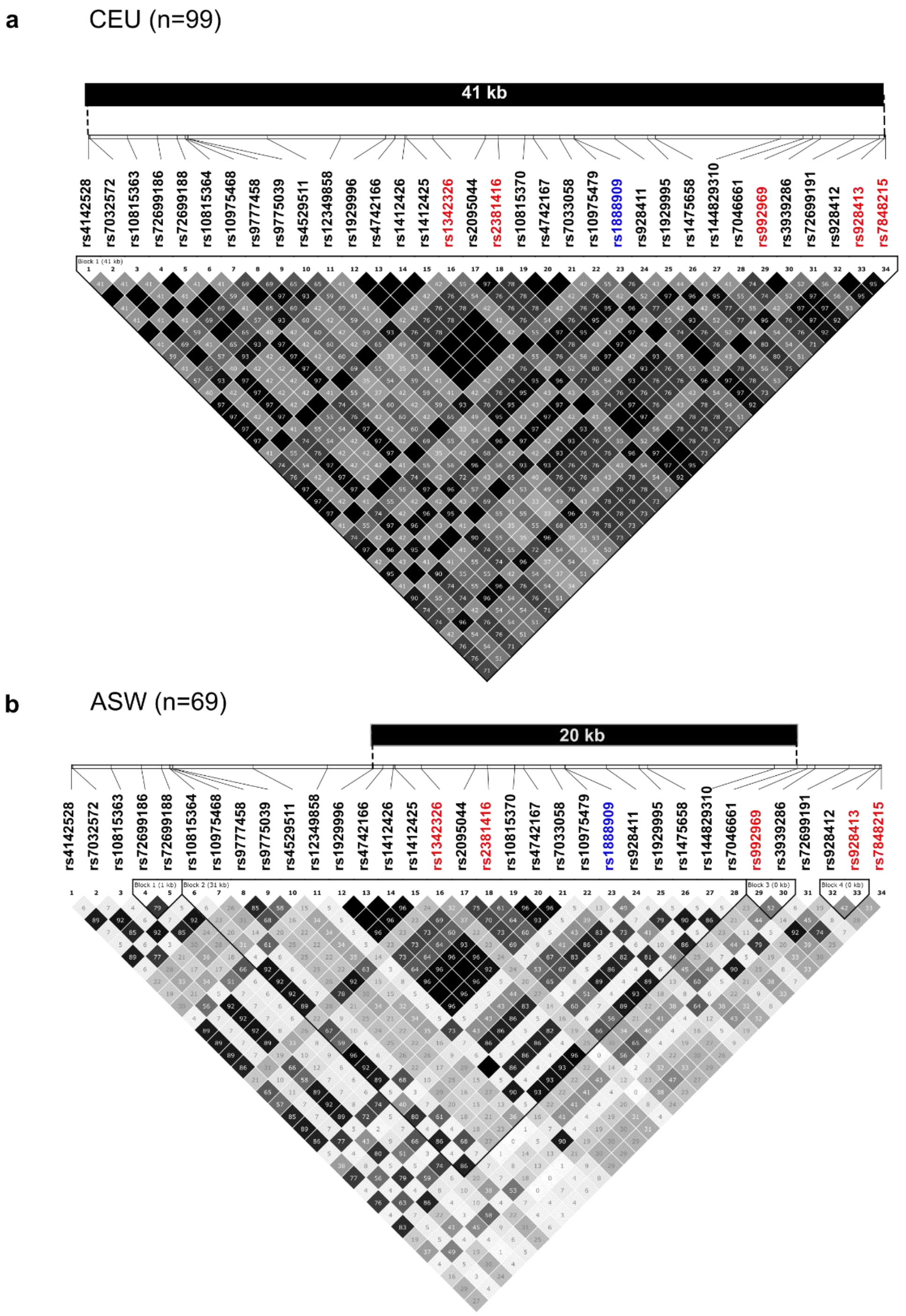

Supplementary Figure 1. 41kb LD blocks created with Haploview software (v4.2) using a MAF=0.1. Genomic coordinates are chr9: 6,172,380-6,213,488 (hg19). Positions of the five lead SNPs (in red) from seven GWAS studies are marked on the Caucasian (CEU) plot. (**a**) and Americans with African ancestry from SW USA (ASW) plots **(b)**. The lead SNP rs1888909 in African Ancestry is shown in blue. The 34 SNPs shown in the plot are either in LD with one of five lead SNPs (in red) in European (r2≥0.8), African ancestry (r2≥0.4) or both populations. (HaploReg v4, https://pubs.broadinstitute.org/mammals/haploreg/haploreg.php)

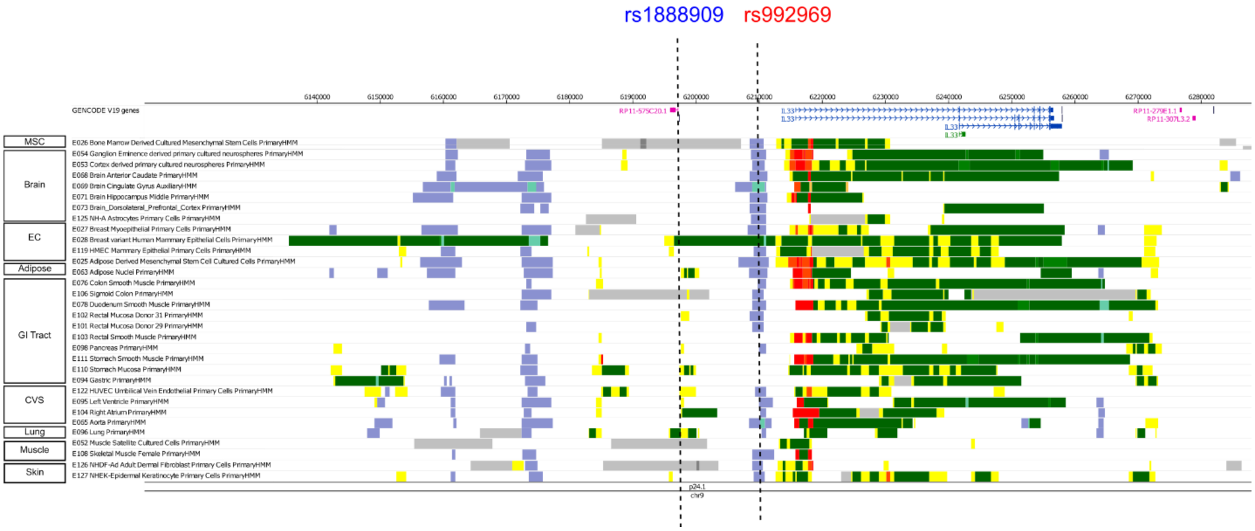

Supplementary Figure 2. Roadmap Epigenome analysis at the *IL33* locus. Rs992969 (in red) was the lead SNP in the combined multi- ancestry GWAS studies, while rs1888909 (in blue) was the lead in the African American/African Caribbean GWAS. Chromatin states from Roadmap Epigenomics Project showing regions with potential regulatory activity- yellow: active enhancer; green: transcription; blue: heterochromatin; red: active TSS; gray: weak repressed polycomb.

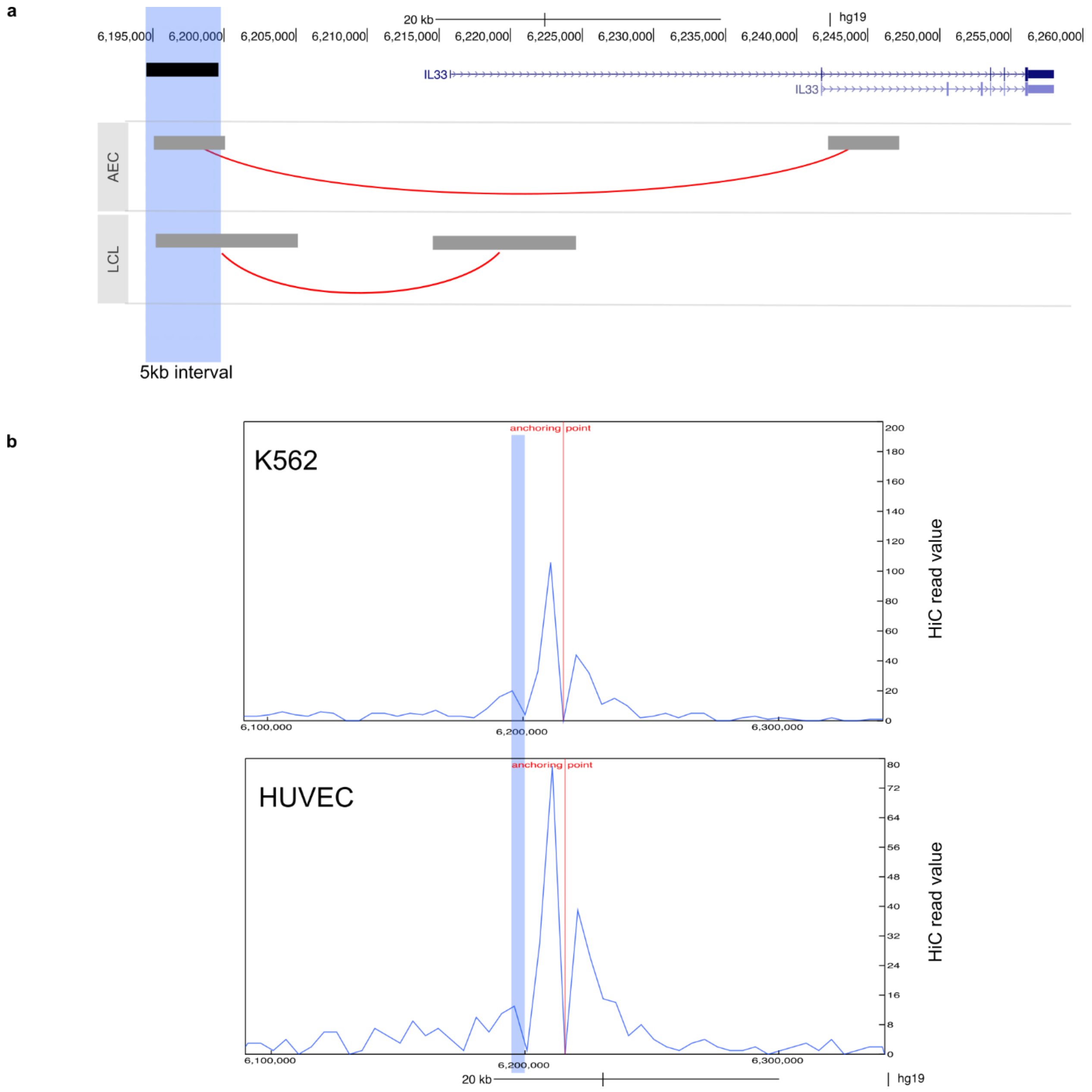

Supplementary Figure 3. Interaction maps show looping of the asthma-associated region to the *IL33* promoter. a Promoter Capture Hi-C from freshly isolated human bronchial epithelial cells (AEC, GEO accession: GSE152550) and human lymphoblastoid cells (LCL; GEO accession: GSE79718; https://www.ncbi.nlm.nih.gov/geo/). Gray Bars correspond to 5kb or 10kb resolution Homer interactions. Arcs depict interactions between the asthma associated 5kb region (blue shaded box) and *IL33* gene. **b** Virtual 4C plots using Hi-C data from myelogenous leukemia cells (K562) and human umbilical vein endothelial cells (HUVEC) from the 3D genome browser (http://3dgenome.fsm.northwestern.edu/). In each plot, the anchoring point (red line) marks the position of the IL33 promoter. Blue shaded box depicts the GWAS- asthma associated 5kb region.

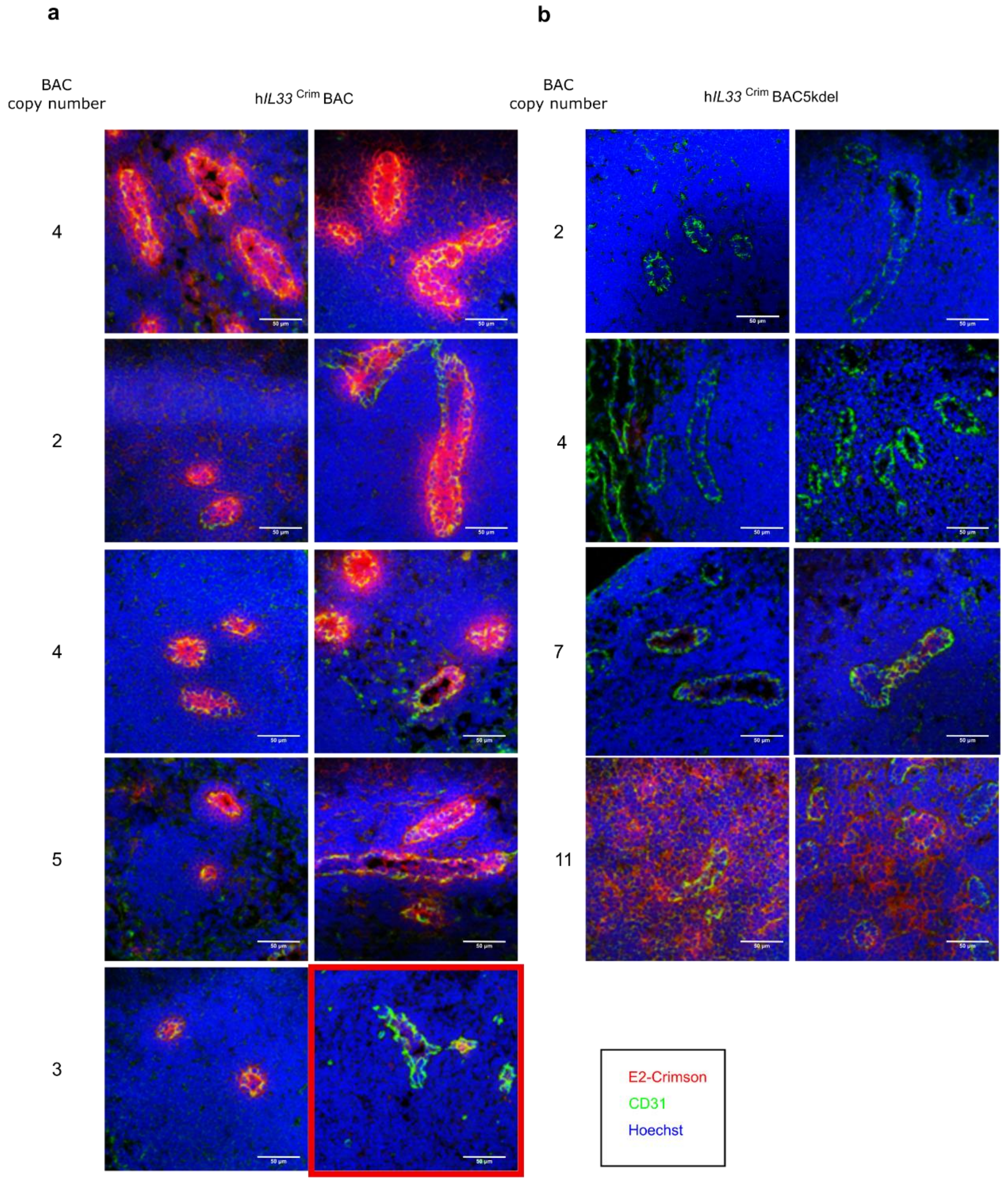

Supplementary Figure 4. Immunohistochemistry of five h*IL33*^{Crim}BAC (a) and four h*IL33*^{Crim}BAC5kdel (b) independent transgenic lines. Estimated BAC copy number is listed adjacent to each founder line. Lymph node sections from two F1 mice for each line are shown. Red indicates Crimson expression. All sections except for one (red box) were stained with αE2-Crimson antibody and illustrate baseline Crimson expression. Green indicates staining with αCD31. Nucleic acid stain Hoechst is blue. Scale bar: 50 μM

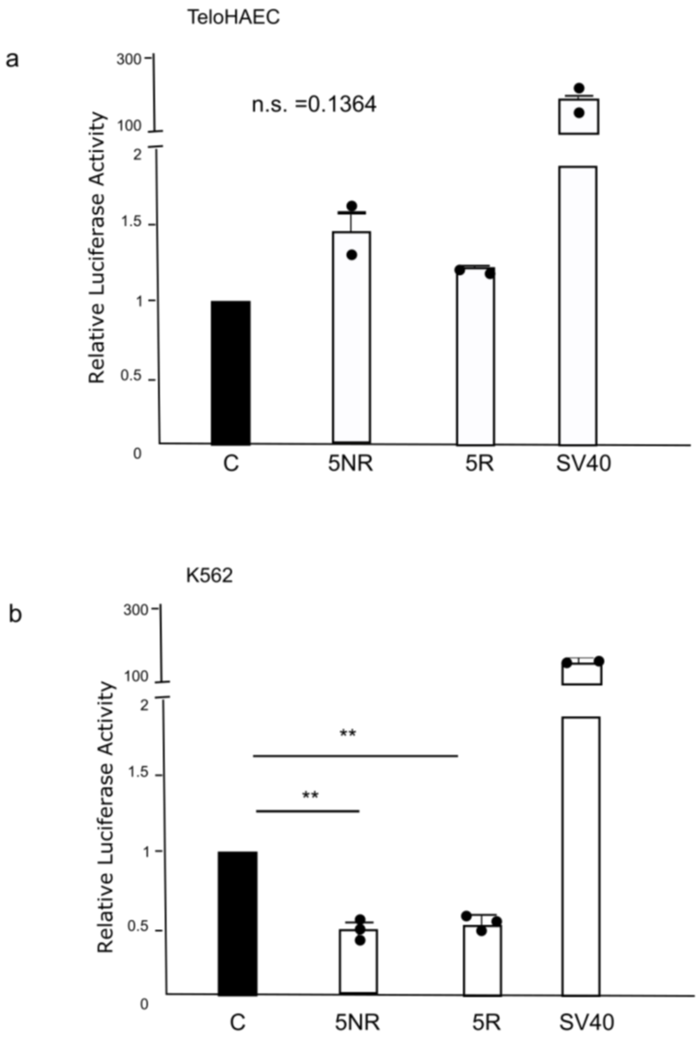

Supplementary Figure 5. Luciferase enhancer assay. Human aortic endothelial cells (TeloHAEC) (**a**) and K562 (**b**) were transfected with a 5kb (chr9: 6,194,500-6,199,500; hg19) construct containing the non-risk (NR) or the risk (R) asthma- associated alleles. As a negative control (scramble), we used a DNA sequence (chr9: 6,161,550-6,162,024; hg19) devoided of any epigenetic mark and as positive control we used PGL3 control vector (SV40; Promega). Firefly luciferase/renilla luciferase provide the normalized luciferase activity. Fold change was calculated relative to the control sequence. Data are presented as mean ± SEM (n=2 independent TeloHAEC experiments; and n= 3 independent DNA preparations examined over 2 independent K562 experiments): **0.047 (C vs 5NR), **0.032 (C vs 5R); one-way ANOVA with post-roc Sidak multiple comparison test. Source data are provided as a Source Data file.

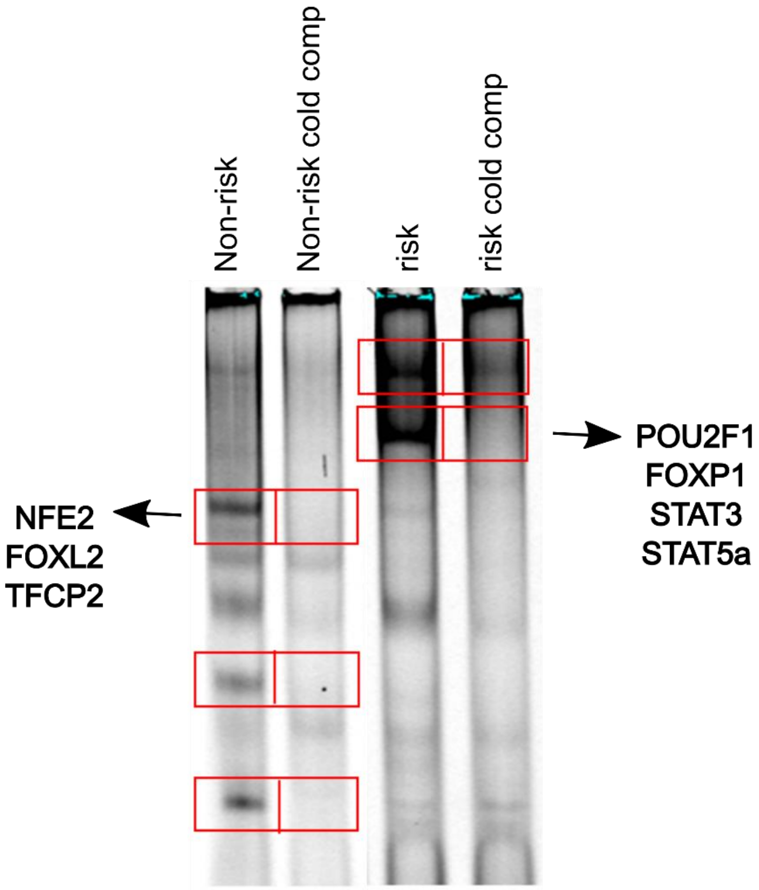

Supplementary Figure 6. **EMSA assay showing outline of bands extracted for mass spectrometry.** Unique transcription factors with a known DNA binding motif are listed next to the boxed bands. Note that no transcription factors were identified in two of the bands from non-risk and one of the bands from risk. Risk and non-risk cold competitor lanes were used as experimental control.

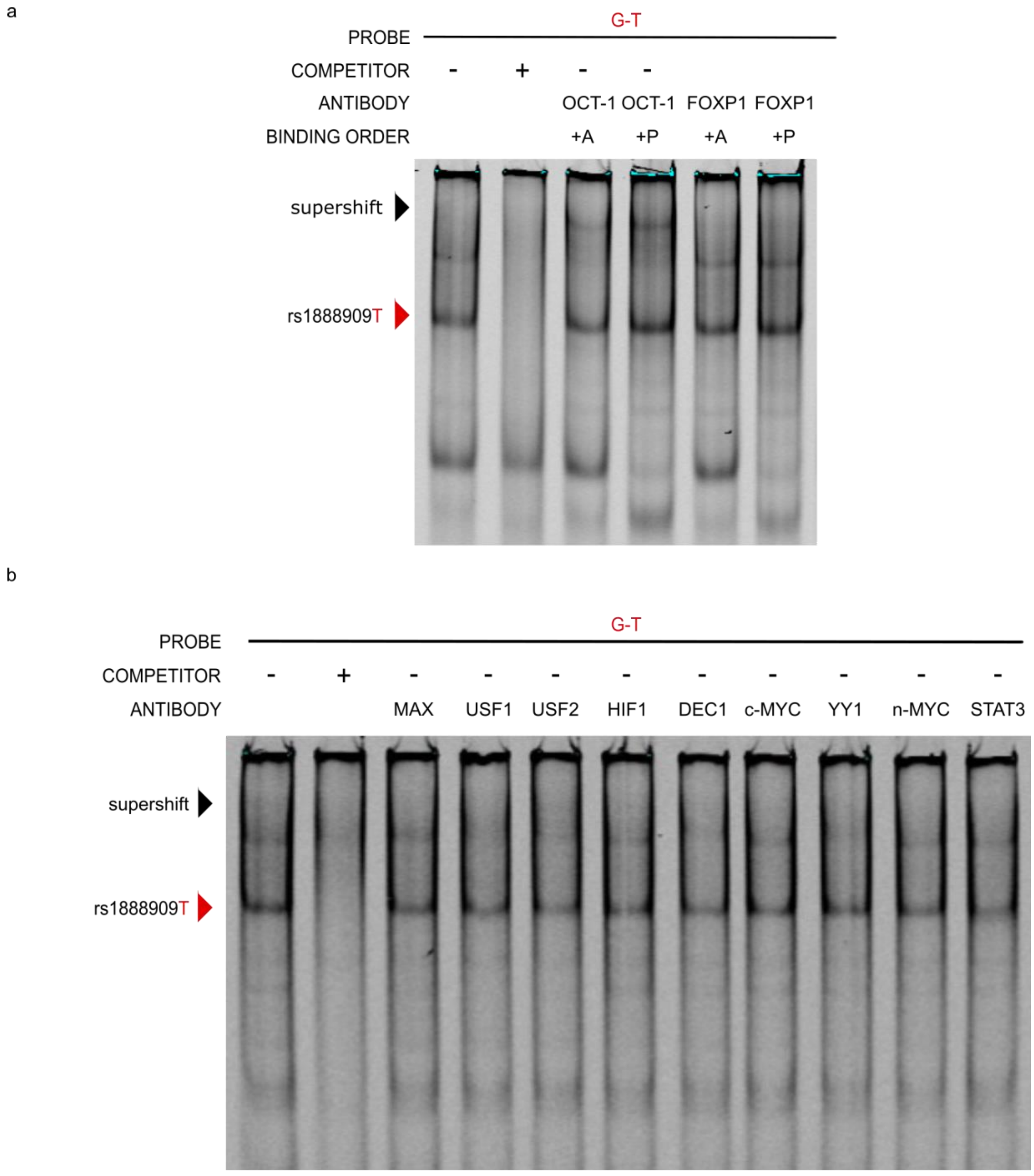

Supplementary Figure 7. EMSA supershift assay. Transcription factors identified by mass spectrometry (a) or predicted by Transfac to bind to alleles of SNPs rs1888909 (b).

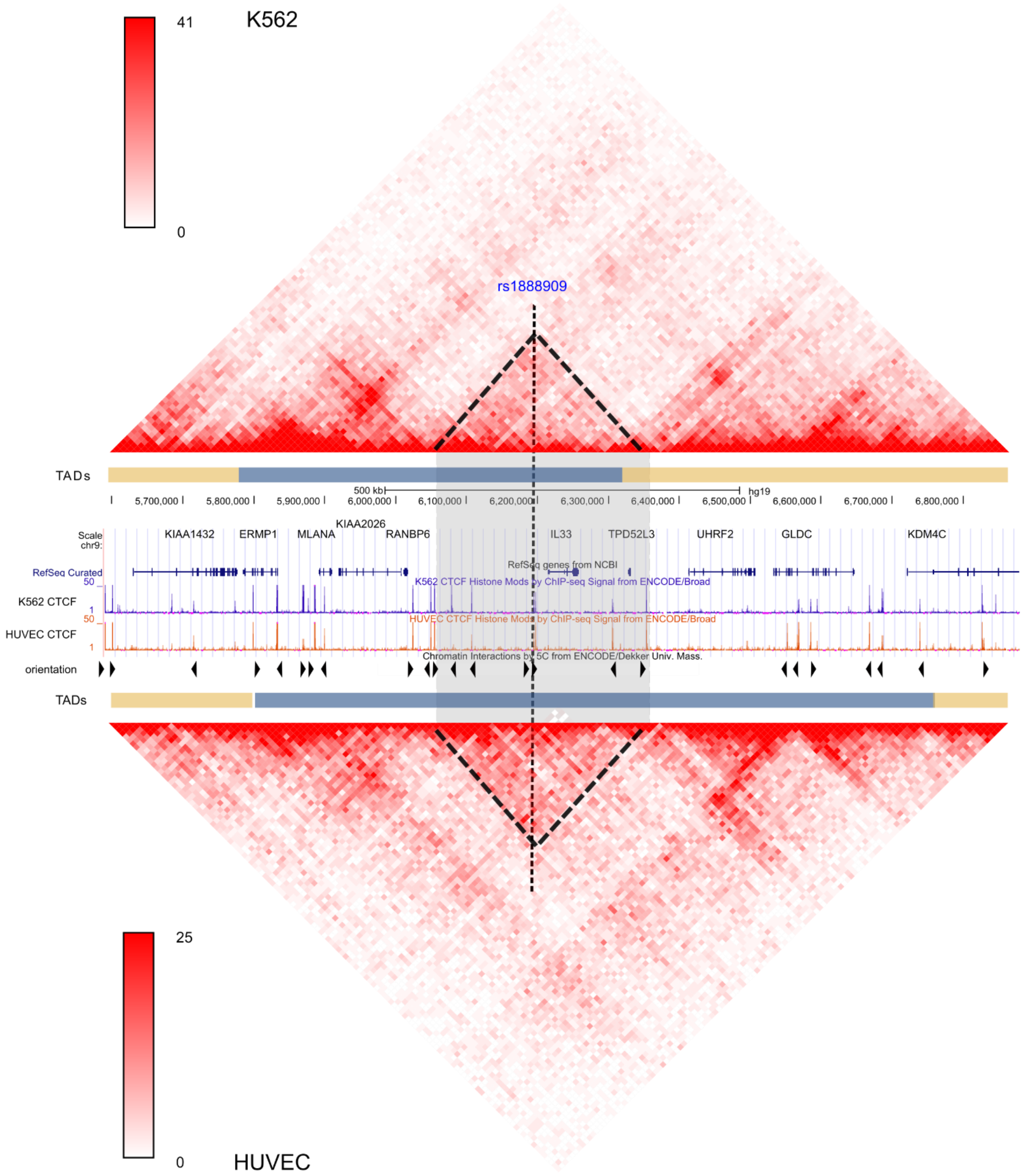

Supplementary Figure 8. TAD structure of the *IL33* locus. A 10-Mb region of K562 and HUVEC Hi-C interaction map at 10kb resolution (hg19; chr9:5590000-6880000.) The alternating yellow and blue bars are predicted TADs. ChIP-Seq tracks for CTCF binding are visualized using the UCSC genome Browser. Black arrow heads show the CTCF orientation. Black dotted line marks a sub-TAD containing the 5kb asthma associated region and the *IL33* gene. Position of SNP rs1888909 (in blue) is marked.

Supplementary Table 1. SNPs in LD ($r^2 \geq 0.80$) with at least one of the five lead SNPs. A total of 26 SNPs spans a 41kb interval in the CEU population. SNPs.

chr	***position (hg19)***	***variant***	***Ref***	***Alt***	***AFR freq***	***AMR freq***	***ASN freq***	***EUR freq***	***GENCODE genes***
9	6172380	rs7032572	A	G	0.15	0.14	0.00	0.16	43kb 5' of *IL33*
9	6175855	rs72699186	A	T	0.16	0.14	0.00	0.15	40kb 5' of *IL33*
9	6176871	rs72699188	C	G	0.14	0.13	0.00	0.15	39kb 5' of *IL33*
9	6177302	rs10975468	C	T	0.15	0.13	0.00	0.15	39kb 5' of *IL33*
9	6177453	rs9775039	G	A	0.30	0.14	0.00	0.15	38kb 5' of *IL33*
9	6178525	rs144511606	G	A	0.15	0.14	0.00	0.15	37kb 5' of *IL33*
9	6185295	rs12349858	C	T	0.07	0.13	0.00	0.16	31kb 5' of *IL33*
9	6187636	rs1929996	C	G	0.28	0.71	0.94	0.72	28kb 5' of *IL33*
9	6188124	rs4742166^	G	C	0.28	0.72	0.93	0.72	28kb 5' of *IL33*
9	6188652	rs1412426^	A	C,T	0.28	0.71	0.93	0.72	27kb 5' of *IL33*
9	6190076	**rs1342326***^	A	C	0.36	0.14	0.00	0.15	26kb 5' of *IL33*
9	6192796	rs2095044^	T	C	0.34	0.76	0.93	0.76	23kb 5' of *IL33*
9	6193455	**rs2381416***^	C	A	0.39	0.77	0.93	0.76	22kb 5' of *IL33*
9	6194831	rs10815370^	C	A	0.27	0.71	0.93	0.72	21kb 5' of *IL33*
9	6195285	rs4742167^	C	T	0.28	0.71	0.93	0.72	21kb 5' of *IL33*
9	6196645	rs7033058^	T	C	0.29	0.71	0.94	0.72	19kb 5' of *IL33*
9	6197377	rs10975479^	A	G	0.08	0.13	0.00	0.15	18kb 5' of *IL33*
9	6197392	rs1888909*^	T	C	0.52	0.78	0.97	0.76	18kb 5' of *IL33*
9	6201163	rs1929995^	T	C	0.07	0.13	0.00	0.16	15kb 5' of *IL33*
9	6208030	rs144829310^	G	T	0.06	0.12	0.00	0.15	7.8kb 5' of *IL33*
9	6209697	**rs992969*^**	A	G	0.68	0.79	0.97	0.76	6.1kb 5' of *IL33*
9	6210099	rs3939286	T	C	0.48	0.78	0.97	0.76	5.7kb 5' of *IL33*
9	6211813	rs72699191	T	C	0.05	0.12	0.00	0.16	4.0kb 5' of *IL33*
9	6213148	rs928412	A	G	0.68	0.79	0.96	0.75	2.7kb 5' of *IL33*
9	6213387	**rs928413***	G	A	0.42	0.78	0.94	0.75	2.4kb 5' of *IL33*
9	6213468	**rs7848215***	C	T	0.58	0.22	0.06	0.25	2.3kb 5' of *IL33*

* lead SNP
^ SNPs defining an LD block of 20kb (chr9: 6,188,124-6,209,099)

Supplementary Table 2. Sum of Single Effects (SuSiE)[22] to fine-map the associated region and identify credible sets (CS) of variants at the *IL33* locus with high probabilities of being causal. CS1 (n=6), CS2 (n=25) and CS3(n=28) are the three CS regions identified.

CS	*SNP*	*position*	*P value*	*PIP*
CS1	rs992969	6209697	6.76E-42	0.391063
CS1	rs1888909	6197392	2.07E-41	0.208944
CS1	rs3939286	6210099	1.86E-41	0.204711
CS1	rs7848215	6213468	7.80E-42	0.085221
CS1	rs928412	6213148	4.38E-41	0.049622
CS1	rs2381416	6193455	1.25E-40	0.030186
CS2	rs1330124	6053098	2.36E-26	0.093386
CS2	rs343495	6069445	2.07E-26	0.088878
CS2	rs343487	6062269	4.54E-26	0.053872
CS2	rs343491	6064640	5.43E-26	0.047065
CS2	rs397505	6076823	1.99E-26	0.043794
CS2	rs343490	6064575	6.01E-26	0.043789
CS2	rs378952	6078146	1.91E-26	0.043609
CS2	rs10975413	6049843	6.49E-26	0.041792
CS2	rs343489	6064299	6.73E-26	0.039977
CS2	rs451974	6076844	2.40E-26	0.038493
CS2	rs340934	6081804	5.25E-26	0.037143
CS2	rs371454	6078614	3.08E-26	0.032018
CS2	rs343479	6054314	1.37E-25	0.028717
CS2	rs189348	6073194	5.39E-26	0.028704
CS2	rs380568	6055531	1.31E-25	0.028074
CS2	rs401834	6078991	3.68E-26	0.027771
CS2	rs343475	6073013	6.15E-26	0.026056
CS2	rs393556	6056468	1.59E-25	0.024546
CS2	rs10975418	6057011	1.73E-25	0.023440
CS2	rs340935	6080998	1.25E-25	0.022409
CS2	rs10975412	6049547	2.51E-25	0.021368
CS2	rs343496	6068077	1.08E-25	0.020070
CS2	rs343499	6059157	2.37E-25	0.019209
CS2	rs343476	6072597	1.18E-25	0.016354
CS3	rs10815391	6240235	5.67E-30	0.074113
CS3	rs10815392	6240236	5.67E-30	0.074098
CS3	rs12339348	6233082	3.72E-30	0.073122
CS3	rs10815393	6240324	4.51E-30	0.070343
CS3	rs7035413	6243119	6.89E-30	0.065363
CS3	rs7038893	6243392	9.09E-30	0.063107
CS3	rs17582919	6233376	7.05E-30	0.060414
CS3	rs17498196	6237547	9.47E-30	0.05379

CS3	rs10975504	6235009	9.17E-30	0.051897
CS3	rs72689561	6238750	1.54E-29	0.047636
CS3	rs78757963	6282511	2.44E-19	0.047079
CS3	rs10975507	6236977	1.65E-29	0.037797
CS3	rs76962799	6187132	1.40E-20	0.035635
CS3	rs7851246	6362365	7.51E-28	0.025082
CS3	rs7027505	6345740	4.08E-28	0.024066
CS3	rs16924428	6351111	6.52E-28	0.023837
CS3	rs10975547	6343945	4.60E-28	0.022612
CS3	rs59606381	6362124	1.38E-27	0.019674
CS3	rs7850988	6335760	6.74E-28	0.018757
CS3	rs72689565	6240953	2.84E-28	0.017263
CS3	rs10975558	6364449	2.35E-27	0.016619
CS3	rs16924356	6331610	7.02E-28	0.01507
CS3	rs10491836	6331421	7.55E-28	0.014958
CS3	rs2169287	6305904	2.05E-28	0.013794
CS3	rs10739094	6333685	1.60E-27	0.013582
CS3	rs72691711	6315489	2.72E-28	0.012367
CS3	rs112935616	6236830	5.44E-28	0.011728
CS3	rs7851000	6314487	2.86E-28	0.011704

PIP: Posterior inclusion probabilities calculated using SuSiE; p Values from *Pividori et al* COA GWAS (logistic regression).

Supplementary Table 3. Transcription factors bound to the risk or non-risk EMSA probes identified by mass spectrometry (see methods). EMSA Probes- (risk) rs10975479 (G): rs188808 (T); (non-risk) rs10975479 (A): rs188808 (C).

Transcription factor	***G-T probe***	***Control***	***function***
STAT3*	1	0	Signal transducer and activator of transcription 3;STAT3;ortholog
STAT5A*	3	0	Signal transducer and activator of transcription 5A;STAT5A;ortholog
POU2F1*	2	0	POU domain, class 2, transcription factor 1;POU2F1;ortholog
FOXP1*	1	0	Forkhead box protein P1;FOXP1;ortholog
NFYC	2	0	Nuclear transcription factor Y subunit gamma;NFYC;ortholog
FUS	3	0	RNA-binding protein FUS;FUS;ortholog
PUF60	6	0	Poly(U)-binding-splicing factor PUF60;PUF60;ortholog
ZFHX4	1	0	Zinc finger homeobox protein 4;ZFHX4;ortholog
CAND1	3	0	Cullin-associated NEDD8-dissociated protein 1;CAND1;ortholog
CNOT1	1	0	CCR4-NOT transcription complex subunit 1;CNOT1;ortholog
HMGB1P1	2	0	Putative high mobility group protein B1-like 1
HMGB2	1	0	High mobility group protein B2;HMGB2;ortholog
TARDBP	3	0	TAR DNA-binding protein 43;TARDBP;ortholog
QKI	1	0	Protein quaking;QKI;ortholog
CTBP1	4	0	C-terminal-binding protein 1;CTBP1;ortholog
ERH	2	0	Enhancer of rudimentary homolog;ERH;ortholog
RBM14	1	0	RNA-binding protein 14;RBM14;ortholog
SND1	2	0	Staphylococcal nuclease domain-containing protein 1;SND1;ortholog
Transcription factor	***A-C probe***	***Control***	***function***
FOXL2*	1	0	Forkhead box protein L2;FOXL2;ortholog
NFE2*	1	0	Transcription factor NF-E2 45 kDa subunit;NFE2;ortholog
TFCP2*	1	0	Alpha-globin transcription factor CP2;TFCP2;ortholog
MYCBP	2	0	C-Myc-binding protein;MYCBP;ortholog
DDI2	2	0	Protein DDI1 homolog 2;DDI2;ortholog
NFYB	1	0	Nuclear transcription factor Y subunit beta;NFYB;ortholog
HMGB1	1	0	High mobility group protein B1;HMGB1;ortholog
CTBP1	3	0	C-terminal-binding protein 1;CTBP1;ortholog
ERH	1	0	Enhancer of rudimentary homolog;ERH;ortholog
RBM14	1	0	RNA-binding protein 14;RBM14;ortholog
SND1	2	0	Staphylococcal nuclease domain-containing protein 1;SND1;ortholog

***** transcription factors with known DNA binding motifs bound only to the risk or non-risk probe

Supplementary Table 4. Transcription factors predicted by TRANSFAC to bind the non- risk (NR) or the risk (R) alleles of SNPs rs1888909 and rs10975479

rs1888909C (NR)	***rs1888909T (R)***	***rs10975479A (NR)***	***rs10975479G (R)***
HIF-1	YY1	En1	TEAD1
c-myc	TFII-I	FoxD3	HOXA5
USF-1 (EBOX)	NFIC	Sox3	Nrf2:Mafk
MAX	Hltf	Sox6	MyoD
ZNF354C	OCT-1	AR	myogenin
ARNT(HIFF)	SMARCA3(RUSH)	SRY	DPB
NMYC (EBOX)		NR3C1	CREMtau
CHREBP (CHRE)		Sox9	
		Sox17	
		Sox2	
		GA-BF	

Supplementary Table 5. Comparison between *IL33* expression and protein levels between genotypes for SNPs rs10975479, rs1888909 and rs992969. Confidence interval (95%CI), *p* values and beta shown for each sample group and genotype were obtained by linear regression

Dataset	***SNP***	***p value***	***beta***	***95%CI***
Endobronchial cells/CAG	rs10975479	0.11	0.12	-0.03-0.26
(mRNA)	rs1888909	0.0072	0.14	0.04-0.24
	rs992969	0.035	0.11	0.008-0.22
Nasal epithelial cells/ URECA	rs10975479	0.040	0.216	0.011-0.640
(mRNA)	rs1888909	6.58 E-6	0.289	0.167-0.411
	rs992969	0.0089	0.180	0.047-0.314
Plasma /Hutterites	rs10975479	0.87	0.09	-0.94-1.12
(protein)	rs1888909	0.0026	1.47	0.52-2.41
	rs992969	0.0073	1.50	0.44-2.57

CAG: Chicago Asthma Genetics Study, URECA: Urban Environment and Childhood Asthma (URECA)

Supplementary Table 6. eQTL analysis in human nasal epithelial cells from a birth cohort of children at high risk for asthma (URECA) showing association between variant rs1888909 and genes in the *IL33* locus. *p* Values and beta were obtained through linear regression. Source data are provided as a Source Data file.

Gene	***p value***	***beta***
ERMP1	0.165	-0.049
KIAA2026	0.115	-0.058
MLANA	0.82	0.041
RANBP6	0.384	0.044
IL33	6.58E-6	0.289
UHRF2	0.864	0.010
GLDC	0.68	-0.047

Supplementary Table 7. List of primers

	For BAC recombneering-
IL33-CrimsonKan_F<br>IL33-CrimsonKan_R	TTGAGACAAATGAACTAATATTATATTTTAATCCAACAGAATACTGAAAAATGGATAGCACTGAGAACGTCA<br>GCGTAAAACATTCAGAGATAACTTAAGTCCTTACTTCCCAGCTTGAAACATCAGAAGAACTCGTCAAGAAGG
IL33InsDEL_F:<br>IL33InsDEL_R:	GCACACCTGTAAGTCTCTGCATTTTGCCACTTATACAACTTCATCTTTGAGTGGCACTTTTCGGGGAAATG<br>AAACTTACATCAAATAAAATCTCAACACAGAATTCATACATGTCAACATACTCGAGGCTAGCTCTAGAAGTC
IL33-Crm5'_F:<br>IL33-Crm5'_R	AGCCACAGTTGTTTCCGTTT<br>TTGAGGTAGTCGGGGATGTC
IL-33-Crm3_F'<br>IL-33-Crm3'_R	ATCGCCTTCTATCGCCTTCT<br>TGTGGAGCAAAAAGTGGTTG
	For EMSA
rs10975479G:rs1888909T:	TCTGATGCAGAACAGCAATGTGTTTTCCATGTGCACTTGGTC
rs10975479G:rs1888909C:	CCTGATGCAGAACAGCAATGTGTTTTCCACGTGCACTTGGTC
rs10975479A:rs1888909T	TCTGATGCAGAACAACAATGTGTTTTCCATGTGCACTTGGTC
rs10975479A:rs1888909C	CCTGATGCAGAACAACAATGTGTTTTCCACGTGCACTTGGTC
Consensus OCT-1_F:	TGTCGAATGCAAATCACTAGAA
Mutant OCT-1_R	TGTCGAATGCAAGCCACTAGAA.
	For Luciferase assay
attB1ins5K_F<br>attB1ins5K_R	GGGGACAAGTTTGTACAAAAAAGCAGGCTGCGCTTACATAGGGTCAGATTC<br>GGGGACCACTTTGTACAAGAAAGCTGGGTTAGATGCAGAGCTCCTGTAGTC
	For qPCR
E2-Crimson_ F<br>E2-Crimson_R	GCCAAGCTGCAAGTGACCAA<br>GCCTTGGAGCCGTAGAAGAA
Ppia_F<br>Ppia_ R	AATGCTGGACCAAACACAAA<br>CCTTCTTTCACCTTCCCAAA
OCT-1 F<br>OCT-1 R	GCCTCTGGTCTCAGTGGATA<br>CTGCTCATAGGAGACACAGTAAAG

F: forward primer (5→3); R: reverse primer (5→3)

Supplementary Table 8. List of antibodies

**Antibody**	**Host species**	**Vendor**	**Cat #**	**Amont/Dilution**
Oct-1 (C-21)	rabbit	Santa Cruz	sc-232x	5 µg ChIP, 2ug EMSA
IgG control	rabbit	Santa Cruz	sc-2027	5 µg ChIP, 2ug EMSA
Biotin anti-mouse CD31 (clone 390)	rat	Biolegend	102404	1:250
CD31 Monoclonal Antibody (clone 2H8)	Armenian hamster	Thermo Fisher	MA3105	1:500
Living Colors DsRed Polyclonal Antibody: anti-E2 Crimson	rabbit	Takara Bio	632496	1:500
Alexa Fluor 488 Streptavidin:	goat?	Biolegend	405235	1:250
Anti- Armenian hamster Alex Fluor 568	goat	AbCam	ab175716	1:1,000
Anti-rabbit Alexa Fluor 633	goat	Thermo Fisher	A-21070	1:1,000
FOXP1		Aviva Systems Biology	ARP32564	2 ug
Max (C-17)	rabbit	Santa Cruz	sc-197x	2 ug
USF-1 (C-20)	rabbit	Santa Cruz	sc-229x	2 ug
USF-2 (C-20)	rabbit	Santa Cruz	sc-862x	2 ug
HIF1a (H-206)	rabbit	Santa Cruz	sc-10790	2 ug
c-MYC (C-20)	rabbit	Novus Biological	NB-600-302	2 ug
DEC1	rabbit	Novus Biological	NB-100-1800	2 ug
YY-1 (C20)	rabbit	Santa Cruz	sc-281	2 ug
n-MYC (9E10)	mouse	Novus Biological	NB-200-109	2 ug
Stat3 (C-20)	rabbit	Santa Cruz	sc-482x	2 ug
