# Supplementary material for: Asthma-associated genetic variants induce IL33 differential expression through an enhancer-blocking regulatory region
Source: Nat Commun. 2021 Oct 21;12:6115. doi: 10.1038/s41467-021-26347-z (PMC8531453; doi:10.1038/s41467-021-26347-z)
Supplement: Supplementary file 3 — Reporting Summary [file 41467_2021_26347_MOESM3_ESM.pdf]

nature research

Corresponding author(s): Ivy Aneas. Anne I. Sperling and Marcelo A. Nobrega

Last updated by author(s): Sep 6, 2021

Reporting Summary

Nature Research wishes to improve the reproducibility of the work that we publish. This form provides structure for consistency and transparency in reporting. For further information on Nature Research policies, see our Editorial Policies and the Editorial Policy Checklist.

Statistics

For all statistical analyses, confirm that the following items are present in the figure legend, table legend, main text, or Methods section.

n/a	Confirmed	
☐	☒	The exact sample size (*n*) for each experimental group/condition, given as a discrete number and unit of measurement
☐	☒	A statement on whether measurements were taken from distinct samples or whether the same sample was measured repeatedly
☐	☒	The statistical test(s) used AND whether they are one- or two-sided<br>*Only common tests should be described solely by name; describe more complex techniques in the Methods section.*
☐	☒	A description of all covariates tested
☐	☒	A description of any assumptions or corrections, such as tests of normality and adjustment for multiple comparisons
☐	☒	A full description of the statistical parameters including central tendency (e.g. means) or other basic estimates (e.g. regression coefficient) AND variation (e.g. standard deviation) or associated estimates of uncertainty (e.g. confidence intervals)
☐	☒	For null hypothesis testing, the test statistic (e.g. *F*, *t*, *r*) with confidence intervals, effect sizes, degrees of freedom and *P* value noted<br>*Give P values as exact values whenever suitable.*
☒	☐	For Bayesian analysis, information on the choice of priors and Markov chain Monte Carlo settings
☒	☐	For hierarchical and complex designs, identification of the appropriate level for tests and full reporting of outcomes
☐	☒	Estimates of effect sizes (e.g. Cohen's *d*, Pearson's *r*), indicating how they were calculated

Our web collection on statistics for biologists contains articles on many of the points above.

Software and code

Policy information about availability of computer code

Data collection	EMSA: LLI-COR Odyssey Imaging System.<br>Mass spectrometry (LC-MS/MS): Thermo Q-Exactive Orbitrap mass spectrometer<br>Mice and ZF transgenics imaging: Leica TCS SP2 laser scanning confocal microscope (Leica Microsystems, Inc.); Leica SP8 laser scanning confocal microscope (Leica Microsystems, Inc.)<br>The midbrain and somites EGFP intensities were quantified using ImageJ (https://imagej.nih.gov/ij/)
Data analysis	Fine Mapping<br>susieR (v0.9.0) was used to caculate the Posterior inclusion probabilities (PIPs)<br><br>Reporter Assays<br>Statistical analysis was performed using GraphPad Prism (v 8.4.3)<br><br>Mass Spectrophotometry and protein identification<br>Tandem mass spectra were extracted and then analyzed by Mascot and X!Tandem algorithms. Scaffold v4.8.4 was used to validate the protein identifications. Peptide identifications were accepted if they could be established at greater than 98% probability to achieve an FDR less than 1.0%.<br><br>Candidate Transcription factor analysis<br>Panther pathway analysis database (http://www.pantherdb.org) was used to filter for DNA-binding proteins. Jaspar (http://jaspar.genereg.net) and Alggen-promo (http://alggen.lsi.upc.es) databases were used to screen these factors for known DNA binding sites. Binding data for random octamers was downloaded from UniProbe (http://thebrain.bwh.harvard.edu/pbms/webworks_pub_dev/downloads.php) and identified all transcription factors that bound at least one octamer containing a risk or non-risk allele.<br><br>Genotyping

Principal components analysis (PCA) was performed using 676 ancestry informative markers included on the genotyping arrays that overlap with the HapMap release 3 using the prcomp function in R (v3.3.3). Genotypes within each genotyping platform and each ethnicity were phased and imputed using the Michigan Imputation Server using the 1,000 genomes phase 3 reference panel.

RNA-seq data : For the RNA-seq data, sample contamination and sample swaps were assess using VerifyBamID v1.1.3. Quality control checks were performed using FastQC. RNA sequences from the bronchial epithelial cells were aligned and annotated to known sequences using the Spliced Transcripts Alignment to a Reference (STAR) version 2.5.3a software. Normalization was performed using the trimmed mean of M-values (TMM) method in R (v3.3.3). Mean-variance trend was adjusted using variance modeling voom (R v3.3.3). PCA was used to determine the effects of known confounding variables on global mRNA expression profiles using the prcomp function in R (v3.3.3).

eQTL/pQTL analysis : For eQTL , voom-transformed gene expression counts were adjusted for age, sex, current smoking status, sequencing pool, and the first three ancestry PCs using the function removeBatchEffect() using the limma package (R v3.3.3). Associations between genotypes at rs1888909, rs992969 and rs10975479 and normalized IL33 gene expression counts were also tested using limma (R v3.3.3), assuming additive effects. 3.3.3), adjusting for sex and age.
For pQTL, associations between genotypes at rs1888909, rs992969 and rs10975479 and IL-33 cytokine abundance were tested using a linear model, assuming additive effects. Genotypes were obtained using PRIMAL50, an in-house pedigree-based imputation tool that imputes variants from whole genome sequences from individuals who were genotyped with an Affymetrix genotyping array.

SNPs in LD with at least one of the 5 lead SNPs were determined by HaploReg v4, https://pubs.broadinstitute.org/mammals/haploreg/haploreg.php. LD blocks were created with Haploview software (v4.2).

For manuscripts utilizing custom algorithms or software that are central to the research but not yet described in published literature, software must be made available to editors and reviewers. We strongly encourage code deposition in a community repository (e.g. GitHub). See the Nature Research guidelines for submitting code & software for further information.

Data

Policy information about availability of data

All manuscripts must include a data availability statement. This statement should provide the following information, where applicable:

- Accession codes, unique identifiers, or web links for publicly available datasets
- A list of figures that have associated raw data
- A description of any restrictions on data availability

Data Availability
All data supporting the findings of this study are available within the article and Supplementary Information file or from the corresponding author upon reasonable request.

Publicly available datasets used in this manuscript:
UniProbe- http://thebrain.bwh.harvard.edu/uniprobe/downloads/All/All_Contig8mers.zip
UKBiobank- https://zenodo.org/record/3248979#.YTTfap5KjUJ
Promoter Capture Hi-C- GSE152550 (AEC) and GSE79718 (LCL; https://www.ncbi.nlm.nih.gov/geo).

Field-specific reporting

Please select the one below that is the best fit for your research. If you are not sure, read the appropriate sections before making your selection.

☒ Life sciences ☐ Behavioural & social sciences ☐ Ecological, evolutionary & environmental sciences

For a reference copy of the document with all sections, see nature.com/documents/nr-reporting-summary-flat.pdf

Life sciences study design

All studies must disclose on these points even when the disclosure is negative.

Sample size

No statistical methodology was used to determine the necessary sample size for the following experiments. However, we decided on replicate number based on previous publications which had success seeing statistical significance for measures of effect similar to what we wished to achieve with these experiments. These replicate numbers were based on the relative variance in the assay as well as the effect size observed

BAC transgenics- 5 independent lines of hIL33Crm BAC (control) and 4 independent lines of hIL33Crm 5Kdel (5 kb asthma associated region deleted) transgenic mice were generated. Each line contains a random integration of the BAC DNA. We selected only the founders (F0) containing intact BAC insertions tested by PCR using primers distributed throughout the BAC. We had 5 control lines and 4 lines containing the deletion. Two F1 animals/ found er line were used for qPCR analysis of E2-Crimson mRNA obtained from lymph node (LN), heart and lung from both BAC strains control (n=10) and deleted (n=6).

Report Assay: For each cell line, two-three technical replicates were performed using different preparations of a given construct. Each construct was transfected in triplicate per experiment.

EMSA : three technical replicates using three different nuclear extracts were performed.

ChIP-PCR: two technical replicates using two different nuclear extracts were performed. Figure 5D shows the data of one experiment.

RNA-seq: from endobronchial brushings obtained from 124 asthmatic and non-asthmatic adult subjects, mostly of European ancestry and from nasal epithelial cell brushings from 189 African American children from high risk asthma families .

IL-33 cytokine levels: measured in plasma from 30 children of European ancestry

Data exclusions	For Crimson mRNA quantification between animals containing the full bac and the 5 kb deletion, one founder line showing abnormal Crimson expression from the 5kb deletion group was excluded from the analyses. This abnormal expression can be related to the very high BAC copy number or positional effect.
Replication	for the cell line studies, reported results were consistent across replicates within the experiment (at least triplicate measurements per sample within experiment) and between replicates (3 independent experiments). For animal studies, measurements were taken in triplicate from 10 biologically independent animals containing the full BAC and 6 biologically independent animals containing the 5 kb deletion. For association studies 3 different cohorts were used to corroborate our hypothesis.
Randomization	All experiments were performed with caution and randomized throughout to reduce potential for batch effects to emerge during analysis. For example, during the RNA-seq analysis, covariates that were included in the model are outlined in the methods section. If the covariate was significantly correlated, adjustment was performed before determining significant differences between groups.
Blinding	The experimental work, data preprocessing and significance calling was conducted by several people depending on the approach used. Each group determined data quality and were made blind to what was expected in each experimental groups or perturbations as much as possible.

Reporting for specific materials, systems and methods

We require information from authors about some types of materials, experimental systems and methods used in many studies. Here, indicate whether each material, system or method listed is relevant to your study. If you are not sure if a list item applies to your research, read the appropriate section before selecting a response.

Materials & experimental systems

n/a	Involved in the study	
☐	☒	Antibodies
☐	☒	Eukaryotic cell lines
☒	☐	Palaeontology and archaeology
☐	☒	Animals and other organisms
☐	☒	Human research participants
☒	☐	Clinical data
☒	☐	Dual use research of concern

Methods

n/a	Involved in the study	
☒	☐	ChIP-seq
☒	☐	Flow cytometry
☒	☐	MRI-based neuroimaging

Antibodies

Antibodies used

Oct-1- Santa Cruz, sc-232x, lot #1615 (discontinued- replaced by:Anti-Oct-1 Antibody (12F11): sc-8024)
IgG -Santa Cruz, sc-2027. lot # K2015
rat anti-mouse CD31:Biotin (clone 390), Biolegend, cat#102404, lot# B186738
rabbit anti-E2-Crimson, Clontech [now Takara Bio USA], cat#632496, lot#1904182
Alexa Fluor 488 Streptavidin, Biolegend, cat#405235
Goat anti-Rabbit IgG: Alexa Fluor 633, Thermo Fisher, cat # A-21071
FOXP1, , Aviva Systems Biology, cat # ARP32564
Max, rabbit, Santa Cruz, cat # sc-197x
USF-1, rabbit, Santa Cruz, cat # sc-229x
USF-2, rabbit, Santa Cruz, cat # sc-862x
HIF1a, rabbit, Santa Cruz, cat # sc-10790
Stat3, rabbit, Santa Cruz, cat # sc-482x
c-MYC, rabbit, Novus Biological, cat # NB-600-302
DEC1, rabbit, Novus Biological, cat # NB-100-1800
YY-1, rabbit, Santa Cruz, cat # sc-281
n-MYC, mouse, Novus Biological, cat # NB-200-109

Validation

-Oct-1- Santa Cruz, sc-232x (has been discontinued and replaced by Anti-Oct-1 Antibody 12F11: sc-8024) Anti-Oct-1 Antibody (12F11) is a mouse monoclonal IgG1 κ Oct-1 antibody, cited in 39 publications. is recommended for detection of Oct-1 of mouse, rat, human and Xenopus laevis origin by WB, IP, IF and IHC(P) https://www.ncbi.nlm.nih.gov/geo/query/acc.cgi?acc=GSM1611105

-Normal rabbit IgG, sc-2027– used as negative control for ChIP. https://www.ncbi.nlm.nih.gov/geo/query/acc.cgi?acc=GSM2481676

-CD31:Biotin (clone 390), #102404- mouse monoclonal antibody
Each lot of this antibody is quality control tested by immunofluorescent staining with flow cytometric analysis. https://www.biolegend.com/en-us/abbreviations

--rabbit anti-E2-Crimson, #632496- DsRed Polyclonal Antibody; far-red derivative of DsRed-Express2 for whole-cell labeling application: Immunocytochemistry; include Monitoring transfection efficiencies and In vivo imaging.Strack, R. L. et al. A rapidly maturing . Biochemistry 48, 8279–8281 (2009).
https://www.takarabio.com/products/gene-function/fluorescent-proteins/fluorescent-protein-plasmids/far-red-fluorescent-proteins/e2-crimson-fluorescent-protein

--FOXP1, Aviva Systems Biology, ARP32564) rabbit polyclonal antibody
application: IHC, WB; https://www.avivasysbio.com/sd/tds/html_datasheet.php?sku=ARP32564_T100

-Max (C-17), # sc-197x rabbit polyclonal; epitope mapping at the C-terminus of Max of human origin. 61 product citations on pubmed. application: WB, ChIP, IF https://www.scbt.com/p/max-antibody-c-17

- USF-1 (C-20), sc-229; rabbit polyclonal; epitope mapping at the C-terminus of USF-1 of human origin. 115 product citations on pubmed. application: EMSA, ChIP and WB. https://www.scbt.com/p/usf-1-antibody-c-20

-USF-2 (C-20), sc862x; rabbit polyclonal; epitope mapping at the C-terminus of USF-2 of mouse origin. 67 product citations on pubmed. application: EMSA, ChIP and WB [has been discontinued and replaced by USF-2 (E-3): sc-518074 https://www.scbt.com/p/usf-2-antibody-c-20

- HIF-1α (H-206) sc-10790; rabbit polyclonal; epitope corresponding to amino acids 575-780 mapping near the C-terminus of HIF-1α of human. 19 product citations on pubmed. application: EMSA, ChIP and WB https://www.scbt.com/p/hif-1alpha-antibody-h-206

-YY1 (C-20) sc-281; rabbit polyclonal; epitope mapping at the C-terminus of YY1 of human origin ; 66 product citations on pubmed. application: EMSA, ChIP and WB . https://www.scbt.com/p/yy1-antibody-c-20

-Stat3 (C-20) sc482x; rabbit polyclonal; epitope mapping at the C-terminus of Stat3 of mouse origin; 438 product citations on pubmed. application: ChIP and WB https://www.scbt.com/p/stat3-antibody-c-20

-n-MYC (9E10), Novus Biological, cat # NB-200-109. mouse Monoclonal ,
Validated Applications: WB, Simple Western, ChIP, ELISA, Flow, Flow-IC, IB, ICC/IF, IHC, IHC-Fr, IHC-P, IP, PLA, S-ELISA
https://www.novusbio.com/products/c-myc-antibody-9e10_nb600-302#reviews-publications

-DEC1, Novus Biological, cat # NB-100-1800; rabbit polyclonal. 24 product citations on pubmed
Validated Applications: WB, Simple Western, ELISA, Flow, IB, ICC/IF, IHC, IHC-P, IP, ChIP, CHIP-SEQ
https://www.novusbio.com/products/dec1-antibody_nb100-1800#reviews-publications

-n-MYC, (NMYC-1), Novus Biological, cat # NB-200-109 24 ; rabbit polyclonal product citations on pubmed.
Validated Applications: WB, ChIP, Flow, GS, ICC/IF, IP, CyTOF-ready
https://www.novusbio.com/products/n-myc-antibody-nmyc-1_nb200-109#reviews-publications

Eukaryotic cell lines

Policy information about cell lines

Cell line source(s)

K562- (ATCC#CCL-243); Organism: Homo sapiens, human; Morphology:lymphoblast; Tissue:Bone; Marrow; DiseaseChronic Myelogenous Leukemia Cml (gift from Dr Steve Kron)

TeloHaec (ATCC CRL-4052) ; hTERT-immortalized cell
Organism: Homo sapiens, human; Cell type:endothelial cell; Morphology: endothelial; Tissue:Heart; Aorta; Disease: Normal (gift from Dr Ana Dirienzo)

H292 (ATCC NCI-H292 [H292]CRL-1848™) Human cells; Organism: Homo sapiens, human; Morphology: epithelial; Tssue: Lung; Disease;:Carcinoma; Mucoepidermoid Pulmonary (gift from Dr Steve Kron)

Authentication

cell lines were not authenticated

Mycoplasma contamination

all cell lines were negative for mycoplasma contamination (tested using Universal Mycoplams Detection Kit cat#30-1012K from ATCC)

Commonly misidentified lines
(See ICLAC register)

no commonly misidentified lines were used in this study

Animals and other organisms

Policy information about studies involving animals; ARRIVE guidelines recommended for reporting animal research

Laboratory animals

-hIL33CrmBAC and hIL33Crm5Kdel transgenics lines (derived from CD1) All mice were housed with food and water ad libitum, temperatures of 65-75°F (~18-23°C) with 40-60% humidity and a 14-hour light/10-hour dark cycle. Adult mice aged >8 weeks were

used for reporter gene analysis.

- One cell-stage zebrafish (Danio rerio) embryos were injected with 3–5 nL of a solution containing 25 nM of each construct plus 25 nM of Tol2 mRNA. Embryos where then incubated at 28°C and EGFP expression was evaluated 24 hpf.

Wild animals

no wild animal were used in this study

Field-collected samples

no field-collected samples were used in this study

Ethics oversight

Procedures were conducted with approval of the Institutional Animal Care and Use Committee (IACUC) of University of Chicago (ACUP-71656; IBC0934). in accordance with standard protocols approved by the University of Chicago.

Note that full information on the approval of the study protocol must also be provided in the manuscript.

Human research participants

Policy information about studies involving human research participants

Population characteristics

Genetic fine-mapping of the IL33 locus for childhood-onset asthma in individuals from the UK Biobank. We used the same inclusion criteria and genotype QC measures as reported for Pividori et al. Childhood-onset asthma was defined as onset < 12 yo (n= 9432) and controls as having no reported asthma at latest age of study (n= 318167).

for eQTL:
- 124 adults; males and females (75 European American, 45 African American, 4 other,) with and without asthma (83 cases, 41 controls), who participated in a study of asthma in Chicago (CAG); Genotypes: [10975479 : 100 AA, 19AG,5 GG; rs1888909 :51 CC, 53 CT, 20 TT; rs992969 : 81GG, 49GA, 14 AA]

- 187 African American children , gender [92F, 95M] , 71 asthmatic, 98 controls, and 18 unknown]; genotype [Rs10975479: AA 146, AG 39, GG 2;Rs1888909: CC 45, CT 91, TT 51; Rs992969: GG 86, GA 79, AA 22] from a birth cohort of children at high risk for asthma who participated on the Urban Environmental Childhood Asthma (URECA) study.

For pQTL:
28 Hutterite children from South Dakota; boys and girls; age 7-14, genotype [Rs10975479: AA 21, AG 1, GG 5;Rs1888909: CC 13, CT 10, TT 4; Rs992969: GG 10, GA 11, AA 4]

Recruitment

All subjects provided written informed consent. For the study involving children, written consent was obtained from the parents and written assent was obtained from the children.

Ethics oversight

These studies were approved by University Chicago Institutional Review Board.

Note that full information on the approval of the study protocol must also be provided in the manuscript.
